# Effect of cognitive stimulation workshops on the self-esteem and cognition of the elderly A pilot project

**DOI:** 10.1590/1980-57642018dn12-040013

**Published:** 2018

**Authors:** Thais Sisti De Vincenzo Schultheisz, Regiane Ribeiro de Aquino, Ana Beatriz Ferreira Alves, André Luis Maiera Radl, Antonio de Pádua Serafim

**Affiliations:** 1 Estudante de Doutorado no Programa de Pós-Graduação em Psicologia da Saúde, Universidade Metodista de São Paulo, São Bernardo do Campo, SP, Brazil; 2 Estagiária na Unidade de Neuropsicologia do Instituto de Psiquiatria – HCFMUSP, SP, Brazil; 3 Estudante na School of Arts and Science, University of Miami – USA; 4 Professor do Curso de Fisioterapia da Escola de Ciências Médicas Universidade Metodista de São Paulo, São Bernardo do Campo, SP, Brazil; 5 Diretor da Unidade da Unidade de Neuropsicologia do Instituto de Psiquiatria – HCFMUSP, SP, Brazil; 6 Professor Doutor Colaborador do Departamento de Psiquiatria, Universidade de São Paulo, SP, Brazil; 7 Professor Titular do Programa de Pós-Graduação em Psicologia da Saúde, Universidade Metodista de São Paulo, São Bernardo do Campo, SP, Brazil.

**Keywords:** elderly, cognitive training, memory, attention, neuropsychological rehabilitation, idoso, treinamento cognitivo, memória, atenção, reabilitação neuropsicológica

## Abstract

According to the WHO, by 2025 Brazil will be ranked sixth in the world in terms of proportion of elderly in the population. Within this scope, cognition plays a central role in the aging process, having an important association with quality of life, which suggests the need to develop intervention programs, such as cognitive training.

**Objective:**

To determine the effects of a program of cognitive stimulation workshops on the self-esteem and cognition of elderly people.

**Methods:**

Thirty-eight elderly subjects completed the three-step protocol: 1) Survey of demographic data and evaluation of cognition by a neuropsychological battery and of self-esteem using the Rosenberg Self-Esteem Scale (RSS), prior to training; 2) Participation in twelve cognitive stimulation workshops; and 3) Cognitive evaluation and RSS after the training.

**Results:**

Results showed that the use of training produced positive effects on cognitive test performance of the elderly with and without cognitive impairment. By extension, this demonstrates positive impact on their self-esteem.

**Conclusion:**

These findings encourage investment in cognitive stimulation programs as a resource for improved cognition and quality of life for the elderly. Subjective cognitive complaint may have served as a predictor of decreased self-esteem; therefore, as training improved cognition, it also improved self-esteem.

## RESUMO

Segundo a OMS, em 2025 o Brasil ficará em sexto lugar no ranking mundial em termos de proporção de idosos na população. Nesse âmbito, a cognição desempenha um papel central no processo de envelhecimento, tendo uma importante associação com a qualidade de vida, o que sugere a necessidade de desenvolver programas de intervenção, como o treinamento cognitivo.

**Objetivo:**

Determinar os efeitos de um programa de oficinas de estimulação cognitiva na autoestima e cognição de idosos.

**Métodos:**

Trinta e oito idosos completaram o protocolo de três etapas: 1) Levantamento de dados demográficos e avaliação da cognição por uma bateria neuropsicológica e de autoestima utilizando a Escala de Autoestima de Rosenberg (RSS), antes do treinamento; 2) Participação em doze oficinas de estimulação cognitiva; e 3) Avaliação cognitiva e RSS após o treinamento.

**Resultados:**

Os resultados mostraram que o uso de treinamento produziu efeitos positivos no desempenho do teste cognitivo de idosos com e sem comprometimento cognitivo. Por extensão, isso demonstra um impacto positivo em sua autoestima.

**Conclusão:**

Estes resultados encorajam o investimento em programas de estimulação cognitiva como um recurso para melhorar a cognição e a qualidade de vida dos idosos. Queixa cognitiva subjetiva pode ter servido como um preditor de diminuição da autoestima; portanto, como o treinamento melhorou a cognição, também melhorou a autoestima.

The growth of the elderly population is a worldwide reality and therefore calls for investigations and actions to improve physical and psychological well-being, quality of life and legal capacity among this population.^1^^,^ 2 For example, the presence of subjective cognitive complaints, among other variables, may precede the diagnosis of Alzheimer’s disease.^3^^,^ 4 Cognitive training can reduce impairments due to deficits resulting from brain injuries or neurodegeneration, and can also enhance cognitive functions.^5^^-^ 7 The authors emphasized that elderly people undergoing cognitive training have obtained improvement related to functional connectivity.^8^ This connectivity is promoted by the hierarchical structure of the training, which begins with the most basic attention functions and moves on to more complex ones. Thus, attention incorporates a range of cognitive functions: concentrated, sustained and divided attention, in addition to speed of information processing and consequently improvement in working memory. The study involved 321 elderly subjects, who presented mild to moderate Alzheimer’s disease (AD), mild cognitive impairment (MCI), and no cognitive decline. Participants were divided into two groups: the training group and the control group. Both groups were given cognitive training. The results showed an improvement in the AD evaluation score, and in the functional capacity measured by the scale of instrumental activities of daily living. There were increased short-term verbal hearing and subjective memory complaints in subjects with MCI.^9^ A Brazilian study demonstrated that, after memorizing a list of words, there was an improvement in memory performance, associated with an increase in semantic strategy and activation of the prefrontal cortex of healthy older adults.^10^

The study presents the data from the pilot project involving a program of workshops on cognitive stimulation for the elderly in the ABC Paulista region, with the objective of training cognitive functions: attention, memory, and executive functions. In the present study, we report the results of the workshops on self-esteem and cognition of elderly people with and without cognitive complaints.

## METHODS

### Study design and sample

A cross-sectional study of an intervention in 38 elderly members of social programs in the ABC Paulista region was carried out. Participants were organized into two groups: a Control Group (CG) comprising 20 elderly without cognitive complaints; and a Training Group (TG) containing 18 elderly with self-reported cognitive complaints. The TG was introduced to compare performance between groups.

Inclusion criteria: age 60+, capacity to comprehend verbal commands, and absence of neuropsychiatric disturbances.

### Instruments

Sociodemographic questionnaire.

To evaluate the results of the workshops on cognition and self-esteem, the following instruments were used before and after training:

- Mini-Mental State Examination – MMSE 11 (for cognitive screening).
- WAIS III, Digit span (working memory).^12^
- Trail Making Tests A and B - TMT- (executive functions).^13^
- Rey-Osterrieth Complex Figure Test - ROCFT (executive functions and memory).^14^
- Verbal Fluency-Animal category – VF-A (memory).^15^
- Logical Memory test from the *Wechsler Memory Scale Revised -* WMS-R (memory).^16^
- Rosenberg’s Self-Esteem Scale (RSS) (for evaluation of self-esteem).^17^

### Procedure

The project was introduced in social programs for the elderly and participation was voluntary, involving the following steps:

- Collecting demographic, cognitive and self-esteem data individually.
- Cognitive training: 12 workshops were held for 60 minutes once a week. The activities for each training session are outlined in Table 1.
  **Table 1:** Description of the activities conducted in workshops for both groups.

Workshops	Activities
1) Attention	Fill in the blanks, given the instructions provided, associating spaces and images.
2) Orientation	On a sheet of paper with 72 parallel points, connect the dots guided by a series of arrows
3) Organization	Prepare a menu for breakfast, lunch and dinner from components on a list, without repetition
4) Working memory	Observe a sequence of 8 images for 30 seconds. After removal of the images, reproduce the observed images
5) Reasoning/understanding	Rewrite Roman numeral sentences
6) Organization	Develop a list of varied activities to be carried out from Monday to Friday
7) Attention	Identify misspellings in a list of 40 words in 20 minutes
8) Declarative memory	Write the names of 8 countries, 8 neighborhoods and 5 oceans
9) Working memory	Look at a list of 12 words for two minutes, remove the list and recall as much as possible.
10) Logical-spatial reasoning	Put together a 500-piece puzzle
11) Declarative memory	Create a list containing 35 names of singers (s) or bands
12) Working memory	Watch brief excerpts (4 minutes each) of 5 documentaries or movies. After viewing each excerpt, create a written description of content presented
- One month after the 12th session, all participants were individually reassessed based on cognitive and self-esteem variables.

### Ethics statement

The study was approved by the Ethics and Research Committee of UMESP (CAAE 61808616.0.0000.5508). All participants were required to sign a consent form.

### Statistical analysis

The normality of the distribution of continuous data was verified using the Shapiro-Wilks test, the T-Student´s *t*-test for independent measures. The analysis showed that age and education presented a normal distribution between groups. For the evaluation of the association between groups and the categorical data, Fisher’s exact test and the Chi-Square test were used. To verify differences between groups and between pre and post workshop performance in perceived cognitive and self-esteem, Multivariate ANOVA for estimated marginal means was used. The level of significance adopted was 5% (P<0.05).

## RESULTS

Table 2 shows the sociodemographic data for the sample. A predominance of women can be observed in both groups. No significant differences were found in the evaluation of the association between groups and the demographic data. A similar result was observed for age range of the groups.

**Table 2:** Descriptive sociodemographic data of the 38 participants.

Variables		CG (n=20)			TG (n=18)		p*
		N	%		N	%	
Male		05	25		7	38.9	.715
Female		15	75		11	61.1	
Age (years)	60 to 69	12	60		10	55.6	.194
	Above 70	08	40		8	44.4	
Education	Up to high school	09	45		10	55.6	.214
	Higher education	11	55		8	44.4	
Marital status	Unmarried	3	15		1	5.6	
	Married	9	45		12	66.4	
	Divorced	7	35		0		
	Widowed	1	05		5	28.0	
Age group		**M(SD)**68.6 (7.8)			**M(SP)**69.1 (6.2)		P**.854

M: mean; SD: standard deviation; CG: control group); EG: experimental group. p*: Chi-Square Test and Fisher's Exact Test ); p**: Student´s t-test.

Application of the protocol revealed that self-reported complaints and performance on cognitive tests in the training group were lower than that of the control group (p<.05) (Student´s *t*-test) before the training. The comparison of groups after training demonstrates significant effects on cognitive performance regarding the group effect analysis, time and group interaction * Time in the two groups. The two groups showed improvement on the MMSE, copy and recall of the - ROCFT, an increase in the list of words on the VF-A, an increase in the memory context on the WMS-R and a reduction of the run time on the TMT A and B (p<0.05, test of the estimated marginal means). A similar result was observed in relation to increase in scores on the RSS (Table 3).

**Table 3:** Results of cognitive evaluation and RSS over time, by group effect, time, and group * time interaction.

Variable	Group	T0M±SD	T1M±SD	P Value		
				G	T	G*T
MMSE	CG	26.8±0.91	27.72±0.93	.278	<0.051*	.834
	TG	20.3±1.83	22.3±1.68			
ROCFT-C	CG	29.55±2.32	31.55±2.37	.135	<0.05*	.238
	TG	21.75±2.14	23.8±1.64			
ROCFT-IR	CG	12.5±4.32	13.4±4.51	.091	< 0.05*	.747
	TG	8.4±5.50	10.3±4.93			
VF-A	CG	10.8±1.90	15.5±3.62	.464	< 0.05*	.011
	TG	8.4±5.50	13.4±2.06			
WMS-R	CG	30.6±4.46	31.6±4.67	.512	< 0.05*	.921
	TG	22.2±1.83	24.8±1.89			
TMT-A	CG	40.1±12.05	37.9±10.34	.387	< 0.05*	.354
	TG	58.7±10.06	55.4±9.86			
TMT-B	CG	73.4±19.01	70.8±18.60	.147	< 0.05*	.461
	TG	76.3±18.31	73.1±17.94			
DIGIT	CG	8.7±3.53	9.30±1.83	0.174	0.341	0.816
	TG	7.2±1.55	8.11±1.71			
RSS	CG	25.9±3.53	27.9+±2.28	.588	< 0.05*	.182
	TG	22.2±1.73	24.95 ±2.28			

M: mean; SD: standard deviation; CG: control group; TG: training group); T0: pre-workshop evaluation; T1: post-workshop evaluation; G*T: group*time; MMSE: Mini- Mental State Examination; ROCFT-C: Rey-Osterrieth Complex Figure Test - copy; ROCFT-IR: Rey-Osterrieth Complex Figure Test - immediate recall; VF-A: Verbal Fluency - Animals; WMS-R: Logical Memory test from the Wechsler Memory Scale - Revised; TMT A and B: Trail Making Tests A and B; DIGIT: Digit Span - WAIS III; RSS: Rosenberg Self-Esteem Scale. p<0.05* (* significant interactions, Estimated Marginal Means Test).

## DISCUSSION

This study reports the partial results of a pilot cognitive training program for the elderly population with and without cognitive complaints. This program is the result of a partnership between a university in the ABC region of São Paulo and the psychiatry and neuropsychology unit of São Paulo hospital. The main objective is to verify the effects of this training on cognition, self-esteem and quality of life of this population with a follow up methodology for three years. Our main question is; can cognitive stimulation be established as a protective factor against cognitive impairment in the elderly?

The literature shows that, for cognitive decline caused by normal aging, injury damage, neurodegenerative conditions or subjective complaints, cognitive training has proven effective.^18^^-^ 22

In this study, two groups of elderly people were involved. The training group had self-reported complaints of cognitive deficit, with memory and cognitive performance significantly lower than the control group prior to the training program. Our results showed that the cognitive training, featuring one weekly session for three months, proved capable of promoting changes in the performance in both groups on cognitive exams. Improvement was evident in working memory, for both verbal and visual stimulus (WMS-R and ROCTT). A similar result was observed in the performance of complex visual screening, motor speed, in executive processes, inhibitory control and alternation of attention (TMT A and B). Although we did not assess pathological groups, the improvements on the cognitive tests, in some ways, corroborate the results of previous studies on older populations.^6^^,^ 9 ^,^ 10

How can our results be integrated into literature on the topic of cognitive training? Studies show that the human brain can change in response to lifelong experience. Therefore, the use of cognitive training might be considered a potential resource to improve cognitive decline associated with age.^23^^,^ 24 The evidence of cognitive improvement has been associated with the possible induction of neural plastic changes specific to training on both neural and behavioral levels.^7^^,^ 21 ^,^ 22 ^,^ 24 The literature has emphasized that the possible mechanism for cognitive improvement is related to neural plasticity. The argument for this can be found in studies that emphasize the complex brain’s ability to change in response to both internal demand and external experience.^25^

There was also an increase in the self-esteem after training for both groups. Self-esteem represents the set of feelings and thoughts of the individual with regard to their own value, competence and adequacy; this is reflected in a positive or negative attitude towards oneself.^17^ Subjective cognitive complaints might be a predictor of decrease in self-esteem. Therefore, our results suggest that the perception of better cognitive performance in the CG and TG contributed to an improvement in the self-esteem indexes. These findings corroborate the results of other studies on quality of life, depression and anxiety^26^^,^ 27

Although this is a pilot study, the results corroborate those in the literature.^7^^,^ 22 ^,^ 24 We emphasize that the absence of a follow-up procedure for this study could have produced more consistent data of the effects of cognitive stimulation, representing an *a priori* limitation.

In conclusion, this study demonstrated that the use of cognitive training has positive effects on the cognitive performance of elderly without cognitive impairment, which by extension also positively impacts their self-esteem. The major challenge is to measure evidence of the effects of cognitive training in terms of consolidation over time. These findings support investment in cognitive stimulation programs as a resource for improving cognition and quality of life for the elderly. Therefore, these results are encouraging, warranting the conducting of further studies.
